# A novel *DCTN1* mutation causing perry syndrome leads to abnormal splicing of mRNA: genetic and functional analyses

**DOI:** 10.1007/s13760-023-02368-x

**Published:** 2023-09-05

**Authors:** Wotu Tian, Li Yao, Guochao Shi, Ranran Dai, Li Cao

**Affiliations:** 1 Department of Neurology, Shanghai Sixth People’s Hospital Affiliated to Shanghai Jiao Tong University School of Medicine, Shanghai, 200233 China; 2 Department of Respiratory and Critical Care Medicine, Ruijin Hospital Affiliated to Shanghai Jiao Tong University School of Medicine, Shanghai, 200025 China; 3 Suzhou Hospital of Anhui Medical University, Suzhou, 234000 China

**Keywords:** Insomnia, Depression, Parkinsonism, Hypoventilation, Perry syndrome

To the editor

Perry syndrome (PS, OMIM #168605) is a rare autosomal-dominant neurodegenerative disorder characterized by parkinsonism accompanied by depression/apathy, unexplained weight loss, and rapidly progressive central hypoventilation [1]. In 2009, the Dynactin 1 (*DCTN1*) gene was first identified as the causative gene of PS [2]. However, *DCTN1*-related spectrum ranges widely, additionally including distal motor neuronopathy type VIIB (dHMN7B) and amyotrophic lateral sclerosis (ALS) [3]. dHMN7B presents as weakness and atrophy of distal limb muscle, with early-adulthood or earlier onset and slow progress [4]. Furthermore, *DCTN1*-related-ALS manifests as progressive limb weakness and muscle atrophy, bulbar symptoms, and pathological signs, with onset in the fourth to sixth decades and duration < 10 years. In comparison, *DCTN1*-related-PS is characterized by middle-age-onset, very fast progression (≤ 5 years), the poorest prognosis, and dying of respiratory insufficiency [1].

Herein, we reported a thought-provoking case of a female patient aged 65 years who presented with difficulties in falling asleep and personality changes, progressing within 4 years. Gradually, she experienced shortness of breath at midnight during the last 2 years. Medical recording comprised a 2-year-history of Parkinson’s disease and several episodes of refractory infections and paroxysmal hypoventilation with the need for hospitalization during the last 1 year. Meanwhile, dysphagia, aspiration, and choking occurred frequently. She additionally lost her body weight of 10.5 kg within 1 year. Family history disclosed her mother and two elder sisters with similar symptoms before their death. At the age of 65, she was 160 cm in height and 41 kg in body weight. The patient exhibited a passive attitude and sporadically expressed suicidal ideation. Neurological examination disclosed dysarthria, stiffness, increased muscle tone, and disuse atrophy in the four limbs, with cogwheeling rigidity, tremor, brisk tendon reflexes, and bilaterally positive patellar clonus. The pathological plantar reflex examination wasn’t completed due to unsatisfactory cooperation. In the emergency unit, the arterial blood gas analysis showed increased levels of PaO_2_ (15.73 kPa, normal: 10.5–13.5 kPa), PaCO_2_ (8.06 kPa, normal: 5.1–5.6 kPa), standard residual alkali (9.1 mmol/L) and standard bicarbonate (32.0 mmol/L). During the past 10 months, she had recurrent paroxysmal dyspnea with limb twitching, skin cyanosis, sweating, disordered acid–base balance, suddenly dropped oxygen saturation, and unstable blood pressure. She had experienced respiratory failure and had been bedridden while undergoing intubation-assisted ventilation for several months. Polysomnography (PSG) recording suggested moderate obstructive apnea–hypopnea with severe hypoxemia during sleep. Chest X-ray disclosed complete and partial atelectasis in the left lower and right lower lobes respectively, with left diaphragm elevation (Fig. 1A). Brain MRI showed multiple lacunar foci in bilateral paraventricular and frontal–parietal lobe, brain atrophy, and white matter degeneration (Fig. 1A).

**Fig. 1:**
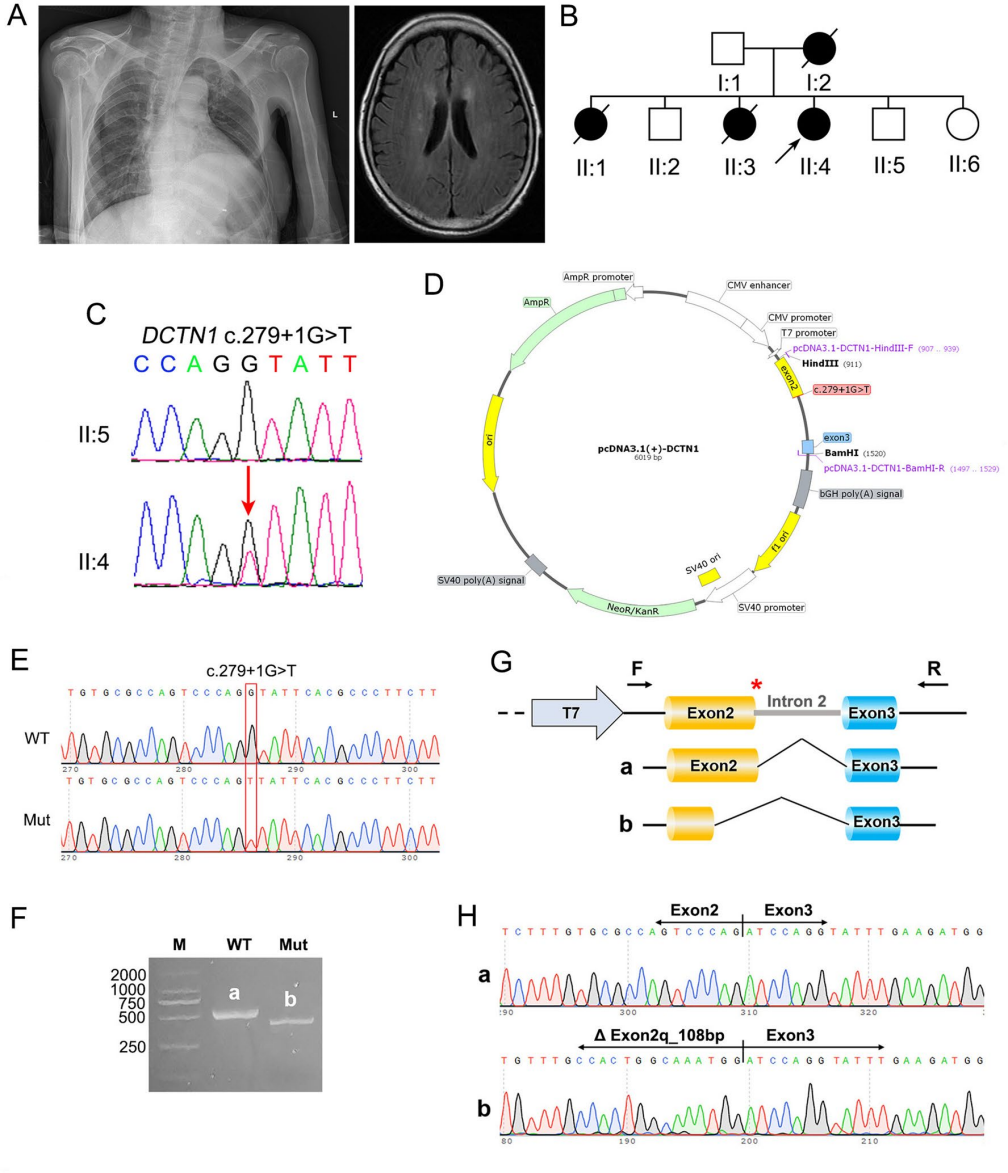
**A** Chest X-ray showing the complete and partial atelectasis of the left lower and right lower lobes respectively, with left diaphragm elevation (left panel). Brain MRI showing multiple lacunar foci in the bilateral paraventricular and frontal parietal lobe, brain atrophy, and mild white matter degeneration (right panel). **B** Schematic diagram of the family pedigree. **C** The *DCTN1* gene mutation and corresponding normal sequence are shown. Sanger sequencing shows one splicing site mutation c.279 + 1G > T of the *DCTN1* gene (arrow), with the proband (II:4) being heterozygous and her unaffected brother (II:5) being negative. **D** Schematic diagram of minigene plasmids of WT or Mut (with variant c.279 + 1G > T). **E** Sequencing diagrams of the recombinant expression vectors. **F** Agarose gel image of RT-PCR fragments (a = WT, b = Mut). **G** Schematic diagram of minigene vector construction strategy (first line) and splicing diagram of WT (**a**) and Mut (**b**). The red asterisk shows the mutant position. **H** The Sanger sequencing of the fragments ‘a’ and ‘b’ from the agarose gel electrophoresis. Band ‘**a**’ was normal with Exon 2 (246 bp)–Exon 3 (79 bp); there is 108 bp missing at the terminal side of Exon 2 in band ‘**b**’, including ΔExon 2 (138 bp)–Exon 3 (79 bp)

Whole exome sequencing disclosed a novel splicing site mutation c.279 + 1G > T in the *DCTN1* gene in the proband (II:4) but negative in her healthy brother (II:5) (Fig. 1B, C). According to the American College of Medical Genetics and Genomics (ACMG) Standards and Guidelines, it was classified as “pathogenic” [4]. The variant c.279 + 1G > T, which is not found in 1000 g, ESP6500, dbSNP, ExAC, gnomAD, or our in-house healthy controls, is predicted to markedly affect splicing by Mutationtaster (disease causing, scored 1.00), SpliceAI (donor loss scored 1.00, donor gain scored 0.74), and Pangolin (splice loss scored 0.87, splice gain scored 0.64). Interestingly, the adjacent variant c.279 + 2 T > C has been previously reported to cause dHMN7B [5]. In functional studies, two minigenes were constructed according to Fig. 1D, comprising mutant/wild type of Exon 2 (246 bp), Intron 2 (273 bp), and Exon 3 (79 bp) of *DCTN1* gene (Fig. 1E). Both mutant and wild type were then transfected into 293 T cells respectively. RT-PCR results (Fig. 1F) showed a band (529 bp) of the wild type in 293 T cells (band ‘a’), and a band smaller than the wild type was found in the mutant group (band ‘b’). The Sanger sequencing was further performed (Fig. 1G), showing that band ‘a’ was normal with Exon 2 (246 bp)–Exon 3 (79 bp); band ‘b’, with 108 bp missing at the end of Exon 2, only included △Exon 2 (138 bp)–Exon 3 (79 bp) (Fig. 1H). In conclusion, the mutant c.279 + 1G > T minigene destroyed the original donor site, leading to 108 bp deletion on the terminal side of Exon 2, thus failing to produce a normal and mature mRNA.

In summary, *DCTN1*-related-PS should be considered in the differential diagnosis of pure hypoventilation in persons without underlying respiratory disease, especially in the presence of emotional changes, unexplained emaciation, parkinsonism, and familial history. Central hypoventilation and refractory respiratory infection are features of the late disease course and are the major cause of sudden death. Sleep disorder can be the earliest symptom, existing among the affected family members for a long time without being investigated. PSG and arterial blood gas analysis should be conducted to evaluate central hypoventilation and to prevent sudden death. Clinicians should raise *DCTN1* gene sequencing as a reasonable strategy during differential diagnosis for patients with familial parkinsonism without known genetic background after the multidisciplinary team consultation.

## Data Availability

The datasets are available from the corresponding author upon reasonable request.
